# Electrospun Nanofiber Mats for Filtering Applications—Technology, Structure and Materials

**DOI:** 10.3390/polym13091368

**Published:** 2021-04-22

**Authors:** Al Mamun, Tomasz Blachowicz, Lilia Sabantina

**Affiliations:** 1 Junior Research Group “Nanomaterials”, Faculty of Engineering and Mathematics, Bielefeld University of Applied Sciences, 33619 Bielefeld, Germany; al.mamun@fh-bielefeld.de; 2 Institute of Physics-CSE, Silesian University of Technology, 44-100 Gliwice, Poland; tomasz.blachowicz@polsl.pl

**Keywords:** air filtration, electrospinning, nanofibers mats, nanofibers

## Abstract

Air pollution is one of the biggest health and environmental problems in the world and a huge threat to human health on a global scale. Due to the great impact of respiratory viral infections, chronic obstructive pulmonary disease, lung cancer, asthma, bronchitis, emphysema, lung disease, and heart disease, respiratory allergies are increasing significantly every year. Because of the special properties of electrospun nanofiber mats, e.g., large surface-to-volume ratio and low basis weight, uniform size, and nanoporous structure, nanofiber mats are the preferred choice for use in large-scale air filtration applications. In this review, we summarize the significant studies on electrospun nanofiber mats for filtration applications, present the electrospinning technology, show the structure and mechanism of air filtration. In addition, an overview of current air filtration materials derived from bio- and synthetic polymers and blends is provided. Apart from this, the use of biopolymers in filtration applications is still relatively new and this field is still under-researched. The application areas of air filtration materials are discussed here and future prospects are summarized in conclusion. In order to develop new effective filtration materials, it is necessary to understand the interaction between technology, materials, and filtration mechanisms, and this study was intended to contribute to this effort.

## 1. Introduction

The atmosphere contains toxic particulate matter (PM) pollutants that are a mixture of particles, microorganisms, spores, toxic gases, heavy metal dusts, and organic pollutants, such as polycyclic aromatic hydrocarbons, benzene, and aerosol particles. In particular, small particles with a diameter of less than 2.5 μm pose a risk of penetrating deep into the bronchioles and human lungs and directly affecting lung breathing. Inhalation of these particles increases teratogenic, mutagenic and carcinogenic risk and leads to lung diseases, such as asthma, stroke, lung cancer, and heart disease [1,2,3,4,5,6,7]. Moreover, air pollutants greatly affect global health, and particulate matter (PM) plays a crucial role among these contaminants [8]. In many areas where air filtration is important, cleaning air and filtering out small air pollutants are very relevant approaches. In biotechnological field, the main requirements are the control of air pollutants, hazardous biological resources and aerosol particles. The automotive and aerospace industries are still looking for the quality and safety of a product aspect to minimize the amount of pollutants. However, the semiconductor industry has already adopted clean room technology to control and reduce product contamination [9,10,11]. In order to achieve high performance air filtration, different materials, including foams, carbon fibers, carbon nanotubes, and different types of fiber filters, such as standard glass, melt blown, cellulose and spunbond fibers, and the mixture of different materials were investigated aiming various air filtration applications [12,13,14,15,16]. Among them, fiber materials have become an important branch of filter materials due to the simplicity of processing and their porous structure. Technically, conventional filtration media like melt-blown and glass fiber cannot capture aerosol fine dust because the pore size of microfibers is relatively large and bacterial filtration is still limited [17]. Hence, these fibrous materials have limited efficiency in filtering the finest particles and are not suitable for all applications because of their large pore size structure and micro-scale fiber diameter [18,19]. Recently, some technologies have been developed to produce nanofibers, such as drawing process, sea-island spinning, and electrospinning, which are more successful in terms of filtering performances and capturing small particles [20,21].

Along with the development of nanofiber production technology, electrospinning is one of the most common and cost effective technologies due to the exceptional properties of nanofibers. The simplicity of use and versatility of using different bio-based and synthetic polymers, as well as blends, offers many advantages over conventional nonwoven manufacturing methods [22,23,24,25]. In addition, the uniformity of nanofibers and nanofiber mat structure with small fiber diameters and the associated ratio of large specific surface area to volume offer further advantages. The nanofiber mats include physical properties, such as high porosity and ultra-thin fiber diameters, as well as advanced chemical properties, such as permeability and porosity, associated with the geometric structure of the fibers [26,27]. Furthermore, it is possible to selectively produce and control the pore size between the nanofibers according to the application definition. In addition, by introducing the antimicrobial agents and particles and changing the morphology of the nanofiber mats into the nanoscale, well-cross-linked pore structure, all the conditions required for trapping fine particles can be met [28,29,30,31,32].

Furthermore, mechanical stability can be enhanced by embedding them in composites or adding more stable layers using 3D printing [33,34]. Other benefits include higher air permeability due to the slip-flow effect of nanofiber mats, which exhibit small pore size and high porosity, and good electrostatic effect, making it very suitable for air filtration applications. Nanofiber mats offer many advantages and have emerged as ideal candidates for suitable and efficient air filtration materials to purify the air. Since they can be effectively and inexpensively manufactured by electrospinning, they offer a promising strategy for efficient air filter materials [35,36,37]. According to the results of several research groups, nanofibers can easily be produced by electrospinning process from natural materials, such as polysaccharides, collagen, silk, cellulose, or synthetic polymers, such as polyacrylonitrile (PAN), poly(lactic acid) (PLA), acrylonitrile butadiene styrene (ABS), polyurethane (PU), polyvinyl alcohol (PVA), poly(ethylene glycol) (PEG), polystyrene (PS), polypropylene (PP), polyethylene terephthalate (PET), polyamide-6 (PA-6), etc. These and many other polymers are well known in the field of filter materials and are applied in air filter applications [38,39,40]. Many different nanofibers with defined properties can also be produced by adding particles and admixtures, and the number of variations is quite impressive. Despite that, many research studies have been committed to improve the air filter materials with high-performance air filters with high filtration efficiency to protect the environment. This is still a major challenge to improve suitable materials for separation and air filtration applications. In this review, we give an overview of the optimized structure, materials and production technology of electrospun nanofiber mats for effective air filter applications.

## 2. Production Methods of Nanofibers Mats

**Figure 1:**
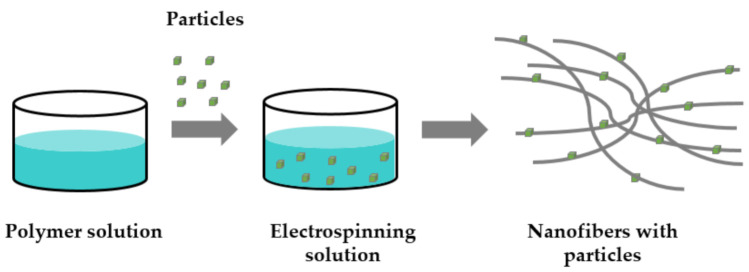
Simple production of nanofibers with particles by adding particles to electrospinning solution.

Interest in electrospinning and nanofiber mats is increasing year by year, and various methods for fabricating nanofibers are described in the literature, including melt electrospinning, coaxial electrospinning, multi-jet electrospinning, needleless electrospinning, bubble electrospinning, electro-blowing, cylindrical porous hollow tube electrospinning, self-bundling electrospinning, and charge injection electrospinning [41]. Among all of these techniques, electrospinning is the most widely used and represents the simplest method for producing nanofibers with a large number of different polymers and admixtures [42,43]. Electrospinning is a simple technology used to produce continuous fine fibers or fiber mats with diameters in the nanometer range. The ultrathin fibers can be made from a variety of materials, such as polymer composites or melts, inorganic or inorganic/organic materials, possibly including ceramics, metallic nanoparticles, particulates, carbon nanotubes, etc., for defined applications [44,45,46,47,48]. Figure 1 shows the production process of nanofibers with particles by simply adding the particles to the electrospinning solution, mixing and producing nanofiber mats with embedded particles.

**Figure 2:**
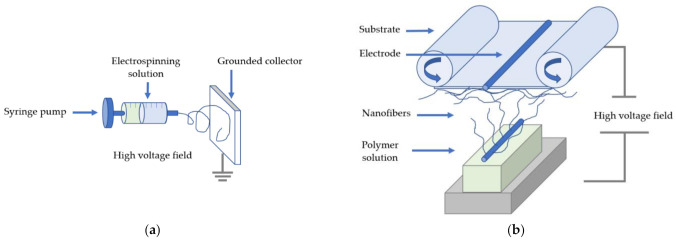
Basic set-up for (**a**) needle based and (**b**) needleless electrospinning (Nanospider).

All electrospinning techniques, such as the needle-based and the needle-free, are based on the same principle (Figure 2). These involve a high electric field applied to a polymer solution, which allows the fibers to form a Taylor cone at the electrode.

The standard needle-based electrospinning systems can be set up in two ways with vertical and horizontal orientation. Due to the working conditions, needleless electrospinning is divided into two types as rotating or stationary spinnerets. The spinnerets connected to a high voltage supply and a dosing unit for the spinning solution play the most important role for fiber quality and productivity. Nanofibers can easily be produced by adjustable control and a polymer solution reservoir (e.g., a syringe with a small diameter). The main parameters for both methods are the solution, the operating and the environmental parameters [49].

Needleless electrospinning is a self-organized process that allows electrospinning of nanofibers directly from an open liquid surface. During the whole process, a thin layer of a polymer solution is built up and rotated on the spinneret surface. This rotation produces conical peaks on the surface of the solution. At the same time, the electrical forces create Taylor cones and create nanofibers.

When preparing the electrospinning solution, the polymers become inserted into a solvent for dissolving and forming polymer electrospinning solution. Most of the polymers used to make nanofibers are dissolved in harmful to highly hazardous organic solvents. Some of them are highly flammable, toxic, harmful to the environment, need special waste disposal, and can cause harm to people, and pose high health and safety requirements [50]. Therefore, there is more demand and interest in “green electrospinning” and low-toxic solvents, such as dimethyl sulfoxide (DMSO), because it is easier to handle and does not cause any problem with disposal. This is why research groups use it. Especially, many researchers are interested in environmentally friendly electrospinning, in which low-toxic solvents and biopolymers are used to produce nanofibers [51,52].

## 3. Summary of the Current Air Filtering Materials

Many different bio-based and synthetic polymers, as well as admixtures, can be used in air filtration applications based on electrospinning technology. With a variety of materials and combinations, such as polymer composites or melts, inorganic or inorganic/organic materials, metallic nanoparticles, particulates, and carbon nanotubes, the diversity of air filtration materials and compositions is almost unlimited [53,54,55,56,57].

### 3.1. Biopolymers and Blends

**Figure 3:**
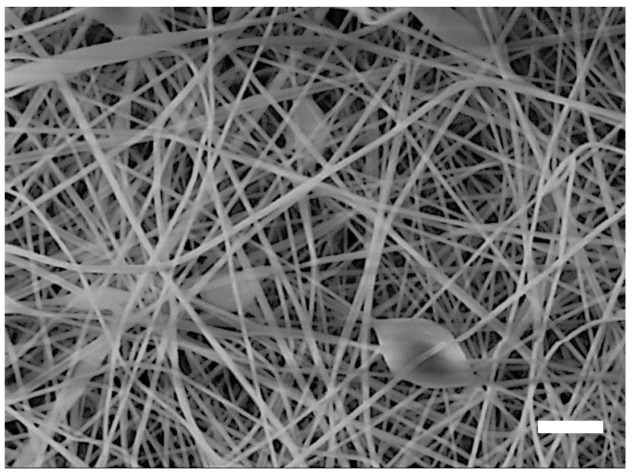
Scanning electron microscope (SEM) image of electrospun 16 wt.% PAN/1.5 wt.% konjac glucomannan nanofiber mat. The bar indicates 2 µm size scale.

Among them, the use of biopolymers offers favorable opportunities from the point of view of environmental protection. Yu et al. fabricated bio-based zein nanofibers that maintain favorable surface morphology even in high humidity environments using chemical crosslinking. A filtration efficiency of over 97% of fine dust size smaller than 0.3 μm was achieved, and the filtration efficiency of other pollutant particles showed efficiency at over 98% [58]. Kadam et al. introduced green electrospun gelatin/β-cyclodextrin (CD)-composite nanofibers from a protein-polysaccharide mixture with excellent filtration efficiency. The prepared gelatin/β-cyclodextrin nanofibers captured aerosols (0.3–5 μm) with <95% filtration efficiency at 0.029/Pa quality factor. In addition, the solution for “green” dual-function breathable air filtration at low resistance was found to exhibit high filtration efficiency [59]. De Almeida et al. synthesized biodegradable and sustainable novel air filters from cellulose acetate (CA) nanofibers and the cationic surfactant cetylpyridinium bromide (CPB), produced by electrospinning technology, to retain aerosol nanoparticles. According to the study, these CA/CPB nanofibers can capture environmental PM2.5, carbon black (BC) and also viruses [60]. Green multifunctional and bio-based chitosan CS/PVA@SiO_2_/Ag NPs air filtration membranes were fabricated, and properties tested at Zhu et al. The filtration efficiency of this multifunctional membrane remained above 96% for particles with a size of 300 nm^−1^ μm (PM10). These nanofiber membranes prove high air filtration performance, biological compatibility and antibacterial properties and offers use as eco-friendly air filtration materials [61]. A biodegradable and multifunctional air filtration nanomembrane was prepared by electrospinning soy protein isolate (SPI)/polyvinyl alcohol (PVA) system at Fang et al., which represents a new type of high-performance environmentally friendly filtration materials. The loading filtration efficiency of the nanofiber membrane reached 99.99% for fine particles smaller than 2.5 μm at low pressure drop. In addition, antimicrobial activity against Escherichia coli was demonstrated [62]. Zhu et al. prepared polyvinyl alcohol (PVA)/citric acid (CA) electrospun nanofibrous membranes via green electrospinning with incorporated hydrophobic silica nanoparticles (SiO_2_ NPs) into the PVA-CA nanofibrous membranes with robust the filtration efficiency [63]. Selatile et al. investigated a depth filtration of airborne agglomerates with electrospun biobased polylactide membranes. The filtration efficiency of >99% of the large particles of 12.07 μm was achieved. The filtration efficiency of the smallest titanium dioxide particles with the particle size of 0.095 μm was 92.97% due to their high penetration ability [64]. Lv et al. reported that multifunctional and bio-based nanofiber mats from chitosan-polyvinyl alcohol blended with silica and silver nitrate nanoparticles (CS/PVA@SiO_2_/Ag NPs) have several superior properties, such as high air filtration performance, biological compatibility, and antibacterial properties [65]. The natural polymer konjac glucomannan (KGM) and pure PVA electrospun nanofiber mats show unique and interesting properties that remove hazardous particles from the air, as Lv et al. report. The filtration efficiency of the ZnO@PVA/KGM membranes for ultrafine particles with the size of 300 nm reached 99.99% [66]. Souzandeh et al. prepared soy protein-based nanofibers with PVA and reported a highly efficient multifunctional air filter material. These SPI-based nanofilters achieved an efficiency of over 98.70% and nearly 100% for PM0.3 and PM2.5, respectively [67]. The further study by Souzandeh et al. investigated the particulate and chemical filtration efficiencies of the gelatin nanofiber mates. A very high filtration efficiency with respect to small particulate pollutants was found. Gelatin based nanofiber mates with an areal density of 3.43 g m^−2^ showed 99.3% and almost 100% removal efficiencies for PM0.3 and PM2.5 pollutants, respectively [68]. Mamun et al. fabricated carbon nanofibers from PAN/konjac glucomannan (KGM) as a precursor by means of needleless electrospinning from the low-toxic solvent dimethyl sulfoxide (DMSO) [69]. These alternative renewable raw materials, such as KGM electrospun with synthetic polymers, reduce environmental impact and offer an alternative to new engineering materials. Figure 3 shows scanning electron microscope (SEM) images of KGM/PAN nanofiber mat.

Liu et al. have studied that zein protein (α-Zein, 95% purity) with 1-butanol, while acetone or ethanol materials can be used to produce nanofiber mats that have very good filtering properties. The filter consisting of a protein functionalized cotton fiber and a protein electrospun nanofiber exhibited filtration efficiencies in capturing particulate pollutants (i.e., PM1.0, PM0.3) above 99.0% for PM of a broad size range [70]. Lv et al. reported that poly(lactic acid) (PLLA)/poly(DL-lactide) (PDLA)/poly(methyl methacrylate) (PMMA) blends show the chemical structure of polymers and the mechanical properties where the air filtration efficiency of PM deposition is increased due to a kind of weak dipolar interaction. In this way, these polylactide stereocomplex (sc-PLA)/PMMA air filters achieved 99.5% PM2.5 removal when 5% by weight of sc-PLA was added to PMMA [71].

### 3.2. Synthetic Polymers

Sundararajan et al. described nylon nanofiber mats which are a potential candidate for filtration applications [72]. The research group of Li et al. produced hybrid PAN/silica (SiO_2_) nanofiber filter composites with desired fiber diameter and pore size, which can effectively remove different particle sizes and it can trap particles with sizes > 0.5 μm through PAN nanofiber mats [73]. Polat et al. reported about nanofiber mates of polyamide 6 (PA6) and polyamide with integrated glass microparticles. The filtration efficiency of these nanofiber mates was 99.97% at a pressure drop of 187.3 Pa [74]. Matulevicius et al.’s study showed that the filtration results from PA6/6 8% (*w/vol*) solutions electrospun nanofiber filter media with the smallest fiber diameters between 62–66 nm proved the highest filtration efficiency between 84.9–90.9% [75].

**Figure 4:**
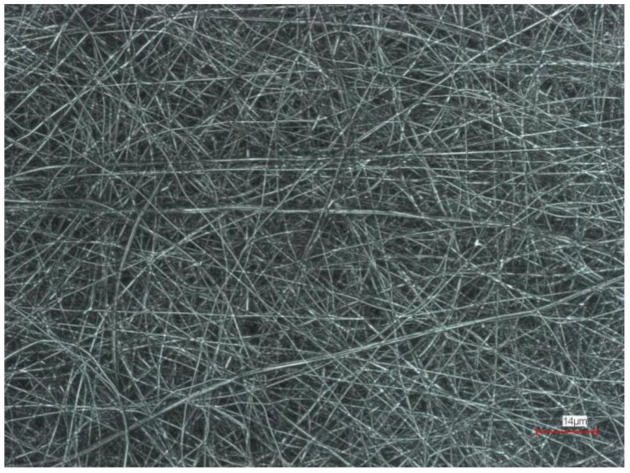
Confocal laser scanning microscopy (CLSM) image of an electrospun 16 wt.% polyacrylonitrile (PAN) nanofiber mat. Scale bar indicates 14 µm.

In Li et al.’s study, nanofibers were fabricated from polycarbonate (PC) and used to filter particulate matter. The results showed that the particulate matter is either trapped by the nanofibers or captured at the surfaces of the fibers by inertial impaction or diffusion. Furthermore, fiber diameter and membrane thickness influenced the filtration efficiency and varied the probability of particles and fiber surfaces colliding [76]. Rao et al. reported that nanofibers-based filter mats are able to capture PM2.5 particles [77]. Canalli Bortolassi et al. produced PAN nanofibers in which various particles were embedded, such as titanium dioxide (TiO_2_), zinc oxide (ZnO), and silver (Ag), in order to study the filtration properties. Their filtration performance was analyzed by sodium chloride (NaCl) aerosol particles with a diameter of 9 to 300 nm using a scanning mobility particle sizer. The highest filtration efficiency with almost 100% achieved TiO_2__F filter but followed with the largest pressure drop (≈183.47 Pa). Ag_F showed high filtration efficiency (>98%) with a low pressure drop of 68.13 Pa. The quality factor of these membranes was higher than that of the commercially available nanofiber membrane for air filtration [78]. Sukitpong et al. applied Ag/TiO_2_ coated on a polyester air filter polyethylene terephthalate (PET) to decrease acetaldehyde, which forms a main toxic stream in cigarette smoke [79]. Atakan et al. have proven polyamide-6/glass nanoparticles to have very good filter properties [80]. Huang et al. varied the PAN concentration of electrospun PAN nanofiber mats to produce a filter and investigated the PM separation efficiency due to the impact of nanofiber diameter, air flow, and nano-bead density on filtration efficiency. The bead-on-string PAN filter achieved a PM removal efficiency of over 99% at a low pressure drop of 27 Pa [81]. A CLSM image of electrospun PAN nanofiber mat is shown in Figure 4.

As Zhou et al. investigated, PAN has excellent properties in terms of chemical stability, abrasion resistance and outstanding weather resistance that can be used in air filter applications [82]. Li et al. demonstrated the filtration performance of commercial air filter media made of electrospun nylon-6 nanofiber mats [83]. Low frequency plasma surface modification of needle punched non-woven polyethylene terephthalate (PET) fibers is a good option for use in filter applications according to Neznakomova et al. [84]. Xiao et al. explained that the air permeability and filtration efficiency of electrospun polyvinylidene fluoride (PVDF) nanofiber mats had positive correlations with pore size [85]. In general, magnetic nanofibers can also be used for filtration by embedding magnetic nanoparticles in a polymer matrix. Kim et al. proposed a novel air filtration system based on magnetic attraction. This filtration system consists of a nanofiber filter (magnetic nanoparticle -nanofiber (MNP-NF)) mixed with magnetic nanoparticles (MNP) [86].

According to Souzandeh et al., more than 95% of PM2.5 hazardous air pollutants can be removed by PAN nanofiber mats in field tests [87]. Leu et al. showed that superhydrophobic poly(methyl methacrylate)/polydimethylsiloxane (PMMA/PDMS) nanofiber mats can transfer water moisture. On the other hand, superhydrophilic chitosan fibers are responsible for a high PM removal [88]. Liang et al. have shown that electrospun thermoplastic polyurethane (TPU) nanofiber mats are very effective in removing high PM2.5 filtration efficiency (99%) [89].

## 4. Air Filtration Mechanisms 

**Figure 5:**
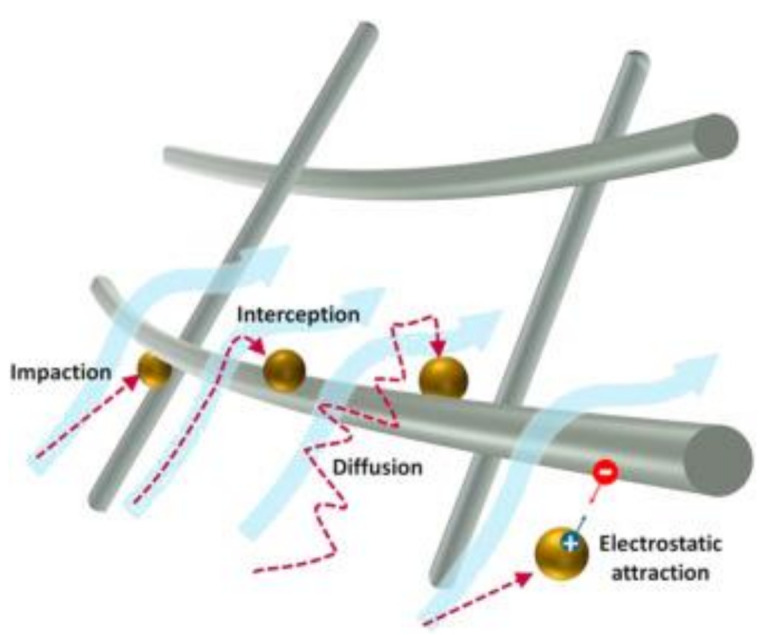
Four major types of particle filtration mechanisms: Impaction, interception, diffusion, and electrostatic attraction. Reproduced from Reference [93], published under a CC BY 4.0 license (Elsevier Ltd.).

The main mechanisms of filtration are based on medium filtration, capturing, deposition, or absorption. Based on natural air filter materials, the filter mechanism can be classified by filtration mechanisms of particulate and gaseous pollutants. The filtration of particles as a function of physical parameters, e.g., fiber diameter, particle size, pore size, and spatial distribution of fibers, air flow, etc., and gaseous pollutants of filtration is mostly controlled by chemical and interaction-based mechanisms [90,91,92]. The capture mechanisms of pollutants on air filters depend mainly on physical and PM size effects. Mechanical filtration mechanisms work by the relative deviation of the particles from the streamlines around the fibers (see Figure 5).

Inertial impaction is based on the inertia of the particles and is the dominant filtration process for relatively large particles that are unable to adjust their direction to the rapidly deforming streamlines. The particles in this case collide directly with the fiber surface [93]. Interception can occur when particles in the streamlines, are not far enough from the surface of a fiber and the radius of the particles are larger than the distance between the corresponding streamline and the fiber surface [94]. When a particle leaves its original path or streamline due to sufficient kinetic energy providing Brownian motion, diffusion begins. As a result, the probability of contact with the fiber surface increases. When electrostatic charges occur between the particles and the filter media, electrostatic filtration mechanism is applied [95].

The sieving mechanism is only relevant for particles whose size is larger than the pore size of the filter. On the other hand, if the pore size of the filter is larger than the particle size, other filtration mechanisms become important, including gravity, elongation, diffusion, inertial shock, separation, electrostatic attraction, and interaction between fiber and contaminant on the surface of the filter [96,97,98,99,100].

For particles smaller than 100 nm, Brownian motion can control the movement for these particles and capture them by random collision with the fibers through the diffusion mechanism [90,101]. The two primary deposition mechanisms for gaseous chemical pollutants are known as physisorption and chemisorption and are based on intermolecular interactions. In physisorption of pollutants, intermolecular interactions between fibers and pollutants are activated at the fiber surface, such as van der Waals interactions, polar-polar interactions, hydrogen bonding, etc. [102,103,104,105]. In chemosorption, a catalytic or non-catalytic chemical reaction occurs on the surface of the fibers and the pollutants are converted into simpler compounds [106].

## 5. Application Areas of Electrospun Air Filtration Media

Electrospun nanofiber mats are promising for many air filtration applications, including biotechnology, pharmaceutical, and indoor air cleaning activities. Electrospun fiber materials prove to have many advantages over conventional air filtration materials. Advantages of nanofiber mats include small diameter, high surface-to-volume ratio, interconnected pore structure and high porosity [107,108]. In addition, the simplicity of manufacturing nanofiber mats and the almost unlimited choice of bio-based and synthetic polymers, as well as integrated particles, provides freedom of many variations. The nanofiber mats, due to their properties, are crucial for numerous applications where the cleanliness of the air is important [109]. The semiconductor industry, where cleanroom air must be free of particles in order not to compromise the quality of products, such as sensors and microchips, filters composed of nanofiber mats appear extremely attractive [110]. In addition, the air in hospitals and laboratories should be constantly filtered and disinfected. Thanks to the simplicity of manufacturing nanofiber mats from various antibacterial biopolymers or the use particles, such as ZnO or silver nanoparticles, can be produced [111,112]. Numerous bacteria, viruses and microorganisms can grow well on moist and warm environment and the air filters are susceptible to microbial colonization [113]. By using antimicrobial nanofibers, the problem could be minimized. Since humans spend a large part of their lives indoors (office, home, school, nursery, etc.), nanofiber mats can be used for air filtration to protect indoor air quality through natural passive ventilation [114]. The indoor air is full of small particles and harmful volatile organic compounds which slowly evaporate from building materials, furniture, flooring, textiles and detergents and cause great harm to people’s health [115,116,117]. Moreover, the air in the cities is getting worse and car tires emit tiny particles that swirl in the air and are constantly carried for kilometers [118]. The electrospun nanofiber mats could prove to be promising automotive interior filters and dust collection materials to protect people’s health. In addition, nanofiber mats are excellent for personal protective equipment, such as protective clothing or face masks. The personal protective equipment with integrated nanofibers is designed to protect from chemical and biological hazards. A mask with nanofibers can have high filtration performance, as well as good breathability, due to its low resistance properties, which makes breathing much more comfortable compared to traditional filtration fibers [119]. The application areas of air filtration materials are various, and future perspectives are promising.

## 6. Conclusions and Future Perspectives

The development of new innovative air filtration materials is of great need due to the air pollution and the increasing number of diseases related to this problem. This study should contribute to the development of effective nanofiber filtration materials and for this purpose, it is essential to understand the interaction between electrospinning technology, materials and filtration mechanisms in order to provide a basis. In this study, a brief overview of the latest electrospun nanofiber mats for air filtration applications is presented, which can support the other research groups in this field. Not only air pollution requires attention, but many industries, such as automotive and aerospace, are striving to improve the quality and safety aspects of products by minimizing the amount of contaminants. Wherever cleanroom technology is needed to control and reduce product contamination, high-performance air filtration is required.

The electrospun nanofiber materials due to their surface-to-volume ratio, pore structure, and ease of manufacture and processing are among the most important materials for improving filtration performance, proving very good filtration performance in terms of fine particles. They have very good filtration efficiency, although they have some limitations, such as the separation of the pollutant particles. The fine electrospun nanofibers enable an increase in the specific surface area of the filter media, resulting in efficient filtration performance of the nanofiber layers. In terms of environmental pollution, more efforts are needed to develop reusable filters and preferably from biopolymers of renewable raw materials and biowaste. The use of bio- and biodegradable polymers helps to protect the environment, because the resources on earth are limited. Apart from that, the use of biopolymers in filtration applications is still relatively not widespread and under-researched. According to research, only a few research groups are currently reporting about bio-based polymers in air filtration applications. This may be because the biopolymers are not always can be electrospun alone and there is a need for a synthetic carrier material, such as PAN or polyethylene oxide (PEO). In addition, biobased polymers have weaker mechanical properties compared to synthetic polymers.

The development of new materials and multi-use air filters would lead to significant cost savings, a reduction in waste, and a positive contribution to the protection of our environment. In addition, the mechanical properties of filters should be further improved to provide comfortable and effective protection against air pollution and against infection or spread of the viral pathogen. This study explains the technology, structure, and application of materials produced by electrospinning technique for use in air filtration. There is a need to constantly control and improve air quality and enforce existing air pollution regulations to protect human health. With the knowledge of this study and continuous development of new materials and methods for air filtration, successful strategies for air filtration could be obtained. New applications and research are needed to develop and implement efficient air filtration materials on an industrial scale.

## Data Availability

No new data were created or analyzed in this study. Data sharing is not applicable to this paper.
